# Ultra-Low Frequency Transcutaneous Electrical Nerve Stimulation on Pain Modulation in a Rat Model with Myogenous Temporomandibular Dysfunction

**DOI:** 10.3390/ijms22189906

**Published:** 2021-09-14

**Authors:** Yueh-Ling Hsieh, Chen-Chia Yang, Nian-Pu Yang

**Affiliations:** 1 Department of Physical Therapy, Graduate Institute of Rehabilitation Science, China Medical University, Taichung 406040, Taiwan; cmusherrie@gmail.com; 2 Kao-An Physical Medicine and Rehabilitation Clinic, Taichung 406040, Taiwan; s901100@gmail.com

**Keywords:** masticatory myofascial pain, parabrachial nucleus, rostral ventromedial medulla, temporomandibular disorder, ultra-low frequency transcutaneous electrical nerve stimulation

## Abstract

Masticatory myofascial pain (MMP) is one of the most common causes of chronic orofacial pain in patients with temporomandibular disorders. To explore the antinociceptive effects of ultra-low frequency transcutaneous electrical nerve stimulation (ULF-TENS) on alterations of pain-related biochemicals, electrophysiology and jaw-opening movement in an animal model with MMP, a total of 40 rats were randomly and equally assigned to four groups; i.e., animals with MMP receiving either ULF-TENS or sham treatment, as well as those with sham-MMP receiving either ULF-TENS or sham treatment. MMP was induced by electrically stimulated repetitive tetanic contraction of masticatory muscle for 14 days. ULF-TENS was then performed at myofascial trigger points of masticatory muscles for seven days. Measurable outcomes included maximum jaw-opening distance, prevalence of endplate noise (EPN), and immunohistochemistry for substance P (SP) and μ-opiate receptors (MOR) in parabrachial nucleus and c-Fos in rostral ventromedial medulla. There were significant improvements in maximum jaw-opening distance and EPN prevalence after ULF-TENS in animals with MMP. ULF-TENS also significantly reduced SP overexpression, increased MOR expression in parabrachial nucleus, and increased c-Fos expression in rostral ventromedial medulla. ULF-TENS may represent a novel and applicable therapeutic approach for improvement of orofacial pain induced by MMP.

## 1. Introduction

Masticatory myofascial pain (MMP) is a regional muscle pain disorder characterized by myofascial trigger points localized in taut bands of masticatory muscles [1], and is also one of the most common causes of chronic orofacial pain in patients with temporomandibular disorders (TMD) [2]. Pain relief is almost at all times the first desire of patients seeking treatment for TMD and poses a challenge for professionals, especially when the pain is chronic and involves other comorbidities and emotional aspects [3]. Ultra-low frequency transcutaneous electrical nerve stimulation (ULF-TENS) is an active therapeutic device that affects relaxation of masticatory and mandibular postural muscles through applying low-frequency, low current stimulation of the mandibular division of the trigeminal nerve and a branch of the superficial facial nerve [4]. According to available literature and the authors’ experience, ULF-TENS seems to be a valid support in the management of TMD patients with more ‘relaxed’ muscles [5,6,7,8,9], but some patients get worse after ULF-TENS, presenting an increase in electromyographic activity [10]. Currently, the mechanisms responsible for the analgesia produced by ULF-TENS remain unclear, especially regarding the involvement of connections in central pain-modulating neurons.

Orofacial pain resulted from TMD may involve the parabrachial nucleus that forms ascending trigemino-parabrachial nociceptive pathways to convey the MMP-induced nociception to higher brain circuits for developing the affective dimension of pain, emotional, and autonomic disturbances [11,12,13]. However, there is growing evidence that the parabrachial nucleus is one of the main connections with the descending pain-modulating systems, best characterized by abundant projections of parabrachial nucleus to the rostral ventromedial medulla involved in pain modulation [14]. An alteration in the descending inhibitory or excitatory influences from some structures such as the rostral ventromedial medulla and central opioid pathway seems to be the most powerful in reducing pain behavior and nociceptive neuronal activity [15]. Therefore, modulation of both parabrachial nucleus and rostral ventromedial medulla that are involved in pain-modulatory circuits can be possible mechanisms behind therapy for MMP.

Substance P (SP) is one such biochemical richly distributed in the parabrachial nucleus and thought to be released from primary afferent terminals by noxious or painful stimuli. Its neuromodulation on transmission in the parabrachial nucleus has been reported [16,17]. Activation of μ-opiate receptors (MOR) in interneurons produces hyperpolarization of neurons, leading to inhibition of firing and modulation of responses to SP, thereby blocking pain transmission [18]. Increased expression of SP in the parabrachial nucleus after tetanic contraction-induced MMP in rat model has been previously identified [19]. In view of these results, this study hypothesizes that ULF-TENS at myofascial trigger points activates neurons in the rostral ventromedial medulla affecting its expression of c-Fos, enhances MOR expression in the parabrachial nucleus, as well as reduces SP expression in the parabrachial nucleus, thus alleviating MMP. Therefore, the aim of this study is to examine the effects of ULF-TENS on electrophysiological activities and functional movements of masticatory muscles, as well as the biochemical alterations in both parabrachial nucleus and rostral ventromedial medulla in animal models of MMP.

## 2. Results

### 2.1. Effects of ULF-TENS on Electrophysiology of Masseter Muscle after MMP Induction

**Figure 1:**
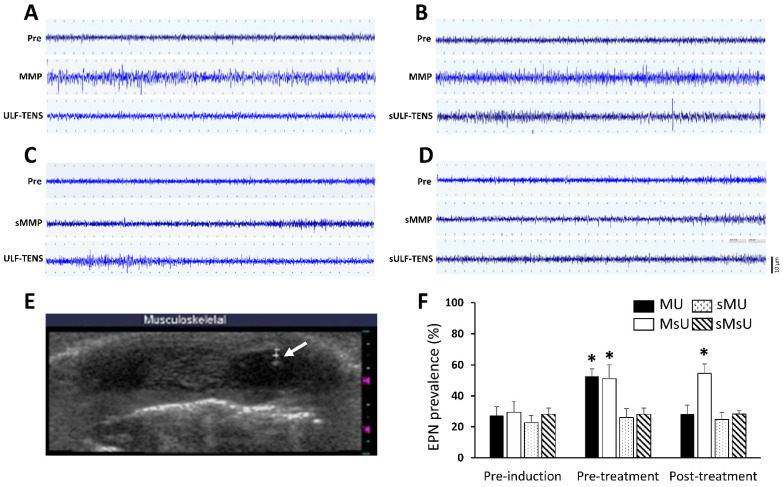
Representative examples of typical EMG activities of EPN from myofascial trigger points of masseter muscle in MU (**A**), MsU (**B**), sMU (**C**), and sMsU (**D**) groups recorded under ultrasonic guidance (**E**). Note that myofascial trigger points are visualized as a hypoechoic region with an elliptical appearance under ultrasound imaging. Alterations of EPN prevalence recorded from masseter muscles at pre-induction, pre-treatment, and post-treatment time points of four groups are shown (**F**). *: *p* < 0.0083 indicates significant differences between either sMU or sMsU group tested by Mann–Whitney test.

Figure 1A–D show serial changes of EPN activities from myofascial trigger points of masseter muscle recorded at the focal hypoechoic area (Figure 1E) under ultrasonic guidance before, after MMP/sham-MMP induction, and after ULF-TENS/sham ULF-TENS treatment in the four groups. Before MMP induction, there was no significant difference in EPN prevalence among the groups (χ^2^(3) = 7.32, *p* = 0.06, Table 1). Significant differences among the four groups were found after MMP induction at both time points of pre-treatment (χ^2^(3) = 29.37, *p* = 0.000002) and post-treatment (χ^2^(3) = 25.87, *p* = 0.00001). After MMP induction, EPN prevalence in both MU and MsU groups were significantly increased compared with that in sMU and sMsU group, indicating marked increase in mean EPN prevalence in masseter muscle after chronic maximum tetanic eccentric contraction (all *p* < 0.0083, Figure 1F, Table 1). After treatment, the MMP-induced increment of EPN prevalence was reduced in the MU group, indicating no statistically significant difference compared with that in sMU and sMsU groups (both *p* > 0.0083, Figure 1F). However, EPN prevalence was still significantly higher in the MsU group than in the other groups (all *p* < 0.0083, Figure 1F). There were significant differences between the MU and MsU groups (Z = −3.82, *p* = 0.00013). Significant difference was found in the difference of improvement from pre-treatment to post-treatment time points between MU and MsU groups (Z = −3.82, *p* = 0.00014, Cohen’s d. = −4.097).

There were significant differences in EPN prevalence among those recorded before induction, before treatment, and after treatment conditions in both MU (χ^2^(2) = 15.79, *p* < 0.017) and MsU (χ^2^(2) = 15.73, *p* < 0.017) groups (Table 1). 

### 2.2. Effects of ULF-TENS on Maximal Jaw-Opening Distance after MMP Induction

**Figure 2:**
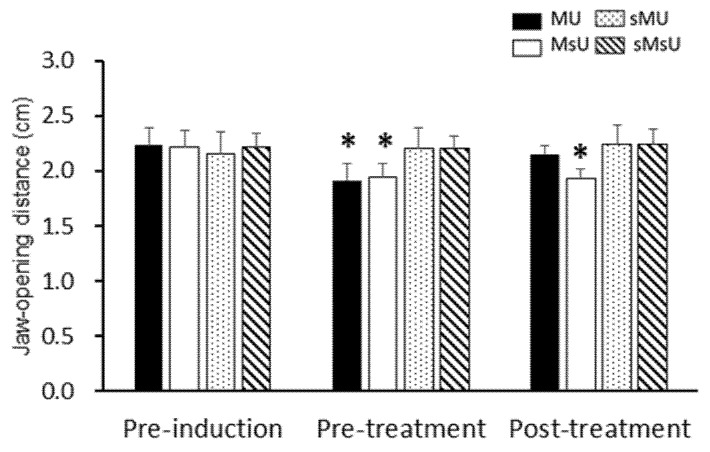
Maximum interincisal distance measured using Vernier caliper in four groups. Data of maximum jaw-opening distance are presented as mean ± SD at the pre-induction, pre-treatment, and post-treatment time points; *: *p* < 0.0083 indicates significant differences between either sMU or sMsU group tested by Mann–Whitney test.

There were significant differences in the maximum jaw-opening distances among those recorded before induction, before treatment, and after treatment conditions in both of MU (χ^2^(2) = 12.60, *p* < 0.017) and MsU (χ^2^(2) = 15.79, *p* < 0.017) groups (Table 1). The maximum jaw-opening distances were significantly decreased in both MU and MsU groups after MMP induction compared with those before induction (both *p* = 0.005, Table 1). However, there were no significant changes after induction in both sMU and sMsU groups when compared with values before induction (*p* > 0.017, Table 1). After treatment, maximum jaw-opening distances were increased in the MU group when compared with those after induction (Z = −2.70, *p* = 0.00687); however, the distances were still significantly more limited in the MsU group than in the other groups (all *p* < 0.0083, Figure 2). Significant difference was found in the difference of improvement from pre-treatment to post-treatment time points between MU and MsU groups (Z = −3.33, *p* = 0.00086, Cohen’s d. = 1.7995).

### 2.3. Expressions of SP-like and MOR-like Immunoreactivity in Parabrachial Nuclei

**Figure 3:**
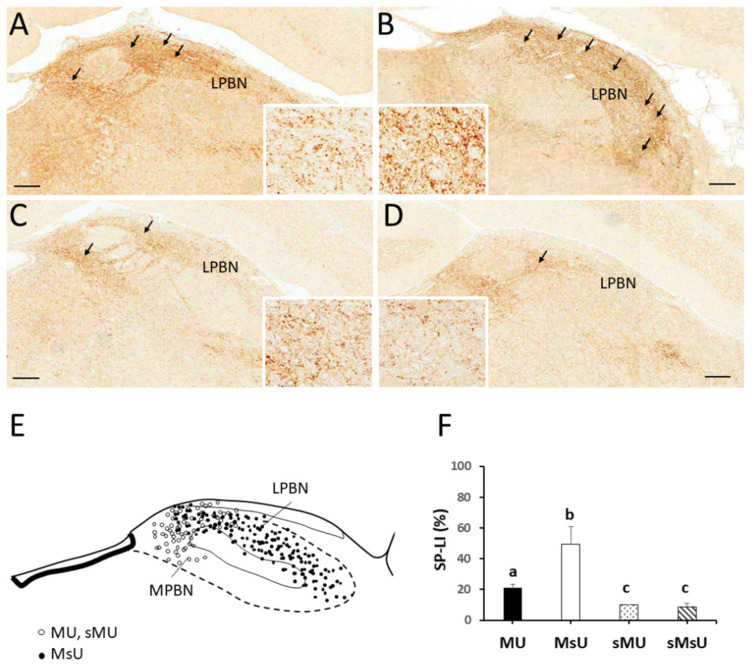
Representative SP-LI staining in sections of parabrachial nucleus (PBN) in rats of MU (**A**), MsU (**B**), sMU (**C**), and sMsU (**D**) groups. The distributions of SP-LI staining area are mostly located in lateral and medial parabrachial nucleus (LPBN and MPBN) in MU, sMU (solid dots), and sMU (open dots) groups (**E**). Data of SP-LI in parabrachial nucleus is presented mean ± SD and values with different superscripts (e.g., a vs. b and b vs. c) indicate significant differences (*p* < 0.0083) for all possible pairwise comparisons of means tested by Mann–Whitney tests (**F**). Scale bars, 250 μm.

Figure 3 shows immunohistochemical expressions of SP proteins in the parabrachial nucleus of each group. Neurons stained with SP-LI were visualized in high-density brown precipitates, along with strong positive pixels of nuclear and cytoplasmic stainings, especially in the lateral parabrachial nucleus. There was higher expression in ventral and internal parts of the lateral parabrachial nucleus in MU rats (Figure 3A). By contrast, the most prominent SP-LI expressed throughout the lateral parabrachial nucleus including ventral, internal, central, superior, and external parts at very high density in MsU rats (Figure 3B). Only sparse expression of SP-LI was found in ventral parts of lateral parabrachial nucleus in sMU and sMsU rats (Figure 3C,D, respectively). Qualitative analysis of SP-LI in the parabrachial nucleus showed different immunoreactivity patterns among the four groups (Figure 3E). Quantitative analysis revealed significantly greater increase of SP-LI in the parabrachial nucleus in the MU and MsU groups than in the sMU and sMsU groups (both *p* < 0.0083, Figure 3F, Table 2). There was significant difference in SP expression between MU and MsU groups (Z = −3.79, *p* = 0.000148, Cohen’s d. = −3.42). 

**Figure 4:**
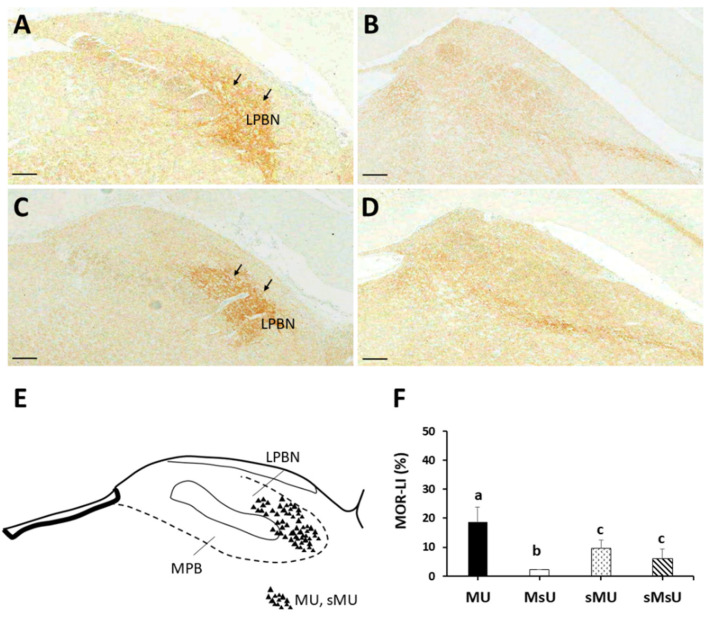
Representative MOR-LI staining in sections of lateral parabrachial nucleus (LPBN) in rats of MU (**A**), MsU (**B**), sMU (**C**), and sMsU (**D**) groups. The distributions of MOR-LI staining area are mostly located in LPBN in MU and sMU groups (triangles, **E**). Data of MOR-LI in LPBN are presented mean ± SD and values with different superscripts (e.g., a vs. b and b vs. c) indicate significant differences (*p* < 0.0083) for all possible pairwise comparisons of means tested by Mann–Whitney tests (**F**). Scale bars, 250 μm.

The most prominent MOR-LI occupied the external part of the lateral parabrachial nucleus at higher density in both MU and sMU rats (Figure 4A,C, respectively). There was only sparse expression of MOR-LI in the lateral parabrachial nucleus in MsU and sMsU rats (Figure 4B,D, respectively). Qualitative analysis of MOR-LI in the lateral parabrachial nucleus showed different immunoreactivity patterns among the four groups (Figure 4E). Quantitative analysis revealed significantly greater increase of MOR-LI in the lateral parabrachial nucleus in the MU and MsU groups than in the sMU and sMsU groups (both *p* < 0.0083, Figure 4F, Table 2). There was significant difference in MOR expression between MU and MsU groups (Z = −3.79, *p* = 0.000152, Cohen’s d. = 4.53). 

### 2.4. Expressions of c-Fos-like Immunoreactivity in Rostral Ventromedial Medulla

**Figure 5:**
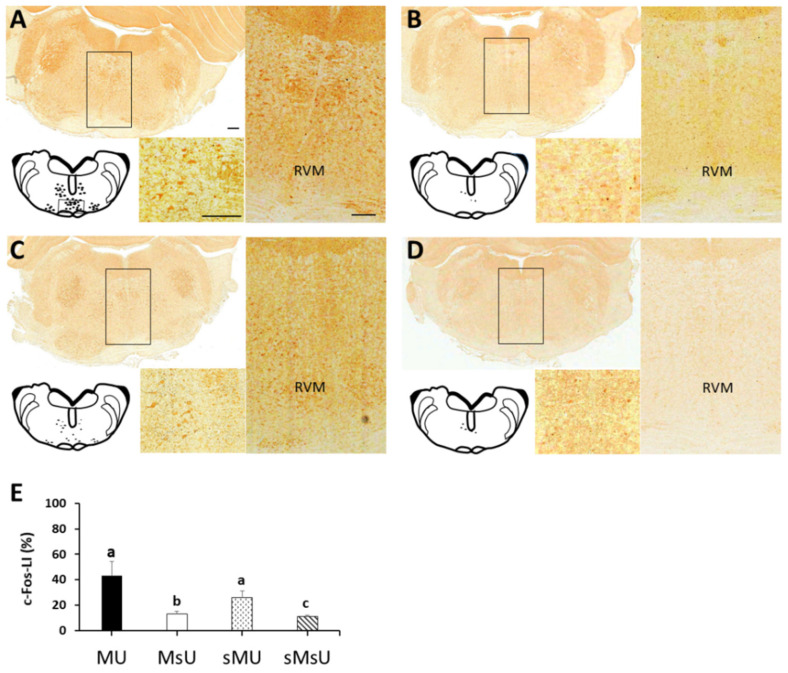
Representative c-Fos-LI staining in sections of rostral ventromedial medulla (RVM) in rats of MU (**A**), MsU (**B**), sMU (**C**), and sMsU (**D**) groups. The c-Fos-LI staining areas are higher in RVM of MU group than of other groups. Data of c-Fos-LI in RVM are presented as mean ± SD and values with different superscripts (e.g., a vs. b and b vs. c) indicate significant differences (*p* < 0.0083) for all possible pairwise comparisons of means tested by Mann-Whitney tests (**E**). Scale bars, 250 μm.

The patterns of Fos-LI noted in the rostral ventromedial medulla area of the four groups after treatments are presented in Figure 5. Qualitative analysis of Fos-LI in the rostral ventromedial medulla showed different patterns of reactivity in the four groups. Fos-LI neurons were visualized as brown precipitates, along with some cytoplasmic staining in MU and sMU groups. Moreover, most of the Fos-LI cells were distributed in the midline nucleus raphe magnus and the adjacent reticular formation ventral to the gigantocellular reticular nucleus in these two groups (Figure 5A,C, respectively). Fos-LI was rarely expressed in neurons of the rostral ventromedial medulla in MsU and sMsU groups (Figure 5B,D, respectively). After ULF-TENS treatment, a significant increase in expression of Fos-LI in the rostral ventromedial medulla was noticed in MU and sMU groups compared with sham ULF-TENS treatment groups of MsU and sMsU groups (all *p* < 0.0083). However, significantly higher c-Fos expression was observed in the rostral ventromedial medulla of the MU group compared with the sMU group (Z = −3.14, *p* = 0.001, Cohen’s d. = 3.91, Figure 5E, Table 2).

## 3. Discussion

In our previous study, repetitive applications of tetanic eccentric contraction in masticatory muscle caused increases in maximum muscle thickness, focal hypoechogenicity in ultrasound imaging, EPN prevalence and amplitudes, and reduction of maximum jaw-opening distance, all indicating successful induction of myofascial trigger points in MMP rats, as well as increased SP expression in the parabrachial nucleus [19]. This study further showed that the application of ULF-TENS would reduce the EPN activity of masticatory muscles at rest, and would increase the maximum jaw-opening distance in MMP rats. It also demonstrated the alterations of MOR- and SP-LI expressions in the parabrachial nucleus as well as increase of c-Fos positive neurons in the rostral ventromedial medulla. These findings were probably related to reduction of nociceptive perception induced by MMP.

TENS has often been employed to alleviate pain in patients with chronic TMD. A previous study found that conventional TENS (high frequency, >50 Hz) and ULF-TENS (<5 Hz) are equally effective in improving acute and chronic masticatory muscle pain and the functional mouth opening [20]. Conventional TENS was effective in improving the functional mouth opening, but the side effects such as tingling sensation and paresthesia occurred in some patients following TENS [20]. There was also significant increase in maximum mouth opening and the maximum bite force after conventional TENS in patients with TMD-related muscle pain, but one patient expressed negative feelings after TENS [21]. Previous evidence has also reported that conventional TENS reduced both pain and EMG activity of the anterior portion of the temporal muscle, increasing the activity of masseter muscles during maximum voluntary clenching in TMD patients. It is possible to conclude that high-frequency TENS (conventional TENS) does not act homogeneously on the features of the electric activity of the muscles evaluated [22]. However, previous evidence demonstrated that ULF-TENS could attenuate movement-evoked pain and improve jaw motor function during repeated jaw movements in patients with TMD and disc displacement without reduction [23]. A retrospective study also demonstrated marked improvement after ULF-TENS along with gradual and progressive disappearance of referred pain symptoms in subjects with craniofacial pain [24]. There is no published study reporting the side effects of ULF-TENS in TMD patients. Previous evidences reported different and complementary analgesic mechanisms when adopting conventional and ULF-TENS with high and low frequencies. High-frequency TENS has been associated with postsynaptic inhibition in the dorsal horn of spinal cord through interrupting nociceptive signals at the spinal cord dorsal horn by stimulating large-diameter low-threshold mechanoreceptive nerve fibers according to the gate control theory of pain [25]. ULF-TENS analgesia may involve a descending inhibitory mechanism that is associated with opioid biochemical transmission and could be partially prevented by spinalization [26]. Therefore, these evidences on the improvement of clinical parameters including reported pain and jaw movement are divergent and controversial depending on the variations in TENS frequency [8,20,22,27]. Furthermore, randomized comparative clinical trials are necessary to optimize the use of TENS (program, duration of sessions, duration of treatment) for TMD.

Many studies have demonstrated the effect of ULF-TENS treatments in normalizing muscle activity and re-establishing the function of masticatory muscles, with no side effects [6,8,24,27]. Surface electromyography (sEMG) activities of masticatory muscles at rest were reduced and muscular activities during clenching were increased in TMD subjects after ULF-TENS treatments for 60 min [6,8]. In the present study, the effects of ULF-TENS on EPN activity, maximal jaw-opening distance, and central nociceptive modulation in rats with myogenous TMD were investigated. Results suggest that ULF-TENS is effective in reducing EPN prevalence of masticatory muscles at rest, increasing jaw-opening distance, and altering nociceptive transmission in brainstems. The electrophysiological and behavioral results are in agreement with those obtained in previous studies [6,22]. Taken together, the present results evidence the effects of TENS on activity of masticatory muscles and support the application of ULF-TENS for TMD pain management.

Findings of several controlled clinical trials indicated that ULF-TENS at the sigmoid notch allows the excitation of motor nerve fibers of the Vth pair of cranial nerves, produces extremely potent reduction of the EMG activity of masticatory muscles associated with relaxation of stomatognathic muscles and increase of interocclusal distance [6,7,8]. A previous study applying ULF-TENS at the intensity inducing contraction of elevator muscles of the jaw showed it is effective in reducing the sEMG activity of masticatory muscles and in improving the interocclusal distance of TMD patients [27]. Accordingly, the results of the present study showed that 15-min ULF-TENS application with intensity inducing a motor contraction of masticatory muscles for seven days led to significant reduction in EPN prevalence of masticatory muscles in rats with MMP-induced TMD. These findings were similar to the electrophysiological results on the mechanism of ULF-TENS. Therefore, it is important for the patient to follow a therapeutic cycle of constant motor contraction of masticatory muscles that must be maintained for the entire session during treatment.

The parabrachial nucleus receives the noxious stimuli from projection neurons in the spinal/trigeminal nucleus, and then sends projection fibers to the higher pain-processing pathway in the central nervous system, which is a functionally and anatomically complex structure involved in nociceptive transmission [28,29]. There is also evidence that the parabrachial nucleus can engage descending pain-modulating systems, the best characterized of which is the rostral ventromedial medulla [14]. These data show that a direct connection from the parabrachial nucleus to the rostral ventromedial medulla conveys nociceptive information to pain-modulating neurons of the rostral ventromedial medulla under basal conditions [14]. Therefore, while the parabrachial nucleus is well known as an important relay for ascending nociceptive information, its functional connection with the rostral ventromedial medulla allows the spinoparabrachial pathway to access descending control systems as part of a recurrent circuit [30]. After ULF-TENS, there were significant alterations in biochemicals of the parabrachial nucleus and increase of Fos-LI in the rostral ventromedial medulla. The descending inhibitory pathways are probably activated to modulate nociceptive transmission after ULF-TENS treatment [31]. 

SP is one of the most important pain-related peptides released from primary nociceptive afferents in response to noxious or painful stimuli, and has a role in transmitting pain in the parabrachial nucleus [17,32]. Activation of opiate receptors in the interneurons produces antinociception and hyperpolarization of the neurons, which may either inhibit the release of SP or reduce the excitatory responses of second-order neurons to SP [18,33]. Parabrachial nucleus containing MOR is likely a site of action for MOR ligands that modulate sensory and/or autonomic aspects of pain transmission in the trigeminal dorsal horn [34]. Evidences of colocalization of endomorphin-2 with SP, calcitonin gene-related peptide, and MOR in primary afferent neurons suggested an interaction of these peptides in pain modulation [35]. Colocalization or shared distribution (overlapping) of these neurotransmitters, or a transmitter and its cognate receptor, may imply an interaction of these elements in the regulation of functions mediated in that region including those that regulate antinociception. Tetanic eccentric contraction of masticatory muscles has been found to increase SP expressions in the parabrachial nucleus, which plays an important role in MMP-mediated chronic pain processing [19]. In this study, ULF-TENS reduced SP expression in the parabrachial nucleus, indicating decrease of nociceptive transmission in rats with MMP induction. This study also found increased expression of MOR in the parabrachial nucleus after ULF-TENS treatment, with SP being predominantly reduced in the external part of the lateral parabrachial nucleus, while MOR is mostly expressed also in the external part of the lateral parabrachial nucleus. It is possible that MOR-containing terminals produce antinociception by decreasing the activity of neurons that receive afferent input from SP-containing axon terminals in the lateral parabrachial nucleus. It is important to note that this is the first report in the published literature on exploring the central modulation of antinociception in the use of ULF-TENS in TMD. Therefore, it may be possible that ULF-TENS may provide an innovative measure on pain reduction in various TMD post-operative complications similar to the previous results of kinesiotapping against orofacial pain induced by molar extraction [36]. 

Some limitations should be taken into account. First, this biochemical study assessed the underlying antinociceptive mechanism of ULF-TENS in MMP-induced TMD only within the brainstem levels (parabrachial nucleus and rostral ventromedial medulla). According to previous studies, action of TENS involves local and central effects. Central activation is thought to occur through endogenous opioid and descending pain modulatory systems, involving the periaqueductal gray and rostral ventromedial medulla regions in animal models with peripheral inflammation [37,38,39]. More advanced studies will be required to reveal a higher central antinociceptive involvement that can provide more information of such neuroplasticity alterations arising from ULF-TENS in management of orofacial pain. Second, this study lacks long-term assessment. Aspects related to fluctuation periods and pain remission in TMD patients must be considered before any final judgment can be made on therapeutic efficacy, which warrants further investigation. Finally, extrapolation of the present conclusion should be cautious because of the absence of evidence for female animals in the measure of interest in the present study. The activity level of female rodents varies dramatically across the estrous cycle, which may introduce a confound on pain behavioral tests that allow animals to move freely [40]. Therefore, most of (at least 79%) animal studies included male subjects only, with a few (near 8%) studies on females only [40]. Further studies are absolutely necessary to determine difference in sex-specific mechanisms of analgesic outcome when ULF-TENS is applied to improve pain management for both sexes.

## 4. Materials and Methods

### 4.1. General Design

**Figure 6:**
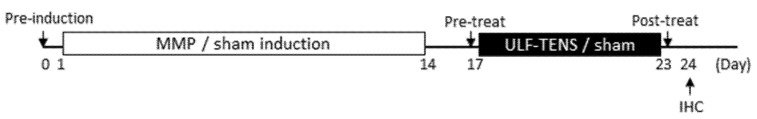
Experimental design. Electrophysiological measurements of endplate noise (EPN), and maximum jaw-opening distance were evaluated. Measurements were obtained before induction (day 0, pre-induction), three days after 14-day MMP induction (before ULF-TENS treatment, day 17, pre-treatment), and after 7-day ULF-TENS treatment (day 23, post-treatment). All animals were then sacrificed on day 24 for substance P, MOR, and c-Fos immunohistochemistry (IHC).

The current experiment was designed to determine the alterations of SP- and MOR-like immunoreactivities (SP-LI and MOR-LI) in the parabrachial nucleus of rats induced by repeated tetanic contraction-induced MMP-like hyperalgesia, and their roles in the generation of these responses after ULF-TENS treatment. Furthermore, rats with diminished MMP characteristics and enhanced c-Fos-LI expression specifically in the rostral ventromedial medulla were also examined. A total of 40 rats were randomly and equally assigned for MMP and sham-MMP induction according to a computer generated randomization list. MMP was induced by 20-min electrical stimulation at the intensity of tetanic muscle contraction for 14 consecutive days. After MMP or sham-MMP induction, animals further received either ULF-TENS or sham-ULF-TENS treatments for seven consecutive days. There were altogether four groups: rats with MMP receiving ULF-TENS (MU group, *n* = 10) or sham treatment (MsU group, *n* = 10), and rats with sham-MMP induction receiving ULF-TENS (sMU group, *n* = 10) or sham treatment (sMsU group, *n* = 10). Manual palpation was performed, maximum jaw-opening distance was measured, electrophysiological recordings were obtained before induction (Day 0, pre-induction), three days after 14-day MMP induction (before ULF-TENS treatment, day 17, pre-treatment), and after 7-day ULF-TENS treatment (Day 23, post-treatment). All animals were then sacrificed on day 24 for immunohistochemistry assessments of SP, MOR and c-Fos proteins. The experimental design and procedures are shown in Figure 6. 

### 4.2. Animal Care and Ethical Approval 

Experiments were performed on adult male Sprague-Dawley rats (SD, 250 to 300 g, purchased from BioLASCO Co., Ltd., Taipei City, Taiwan). The animals were kept on an artificial 12-h light-dark cycle in a university animal center. Food and water were available ad libitum. Each animal was housed individually and cared for following the ethical guidelines of the International Association for Study of Pain in animals [41,42]. Effort was made to minimize discomfort and to reduce the number of animals used. All animal experiments were conducted with procedures approved by the Animal Care and Use Committee in accordance with the Guidelines for Animal Experimentation (No. 2018-059). 

### 4.3. Induction and Identification of Masticatory Myofascial Pain 

The lateral surface of the rat’s face under anesthesia was scrubbed, and secured non-invasively to a horizontal platform in a supine position, with the upper incisors secured to prevent head rotation. MMP can be induced in the masseter muscle of rats by repetitive tetanic eccentric contractions adapted from the forced lengthening technique [43]. Randomly selected unilateral masseter muscles of rats in the MMP group were stimulated to induce maximum tetanic eccentric contraction with jaw closing. The electrical stimulator (Physiomed 3 series EMS, Physio-Med Services Ltd., Derbyshire, UK) was set to deliver a train of supramaximal stimuli at 100 Hz for 300 ms with 250 μs of pulse duration. The cycle was repeated every 30 s with 10 s of rest between stimuli for 20 min per session and for 14 consecutive days. Animals in the sham-MMP group were anaesthetized and the electrode was also inserted into unilateral masseter muscle, but no electrical current was delivered during the same time schedule for their MMP counterparts.

After MMP induction, manual palpation was performed and electrophysiological recordings were made to identify myofascial trigger points in the rat’s masseter muscle for confirming the establishment of a rat MMP model according to the methods published in previous studies [44,45]. Briefly, animal was under anesthesia and its masseter muscle was grasped between fingers from behind the muscle, which was palpated by gently rubbing (rolling) to find a taut band. The palpable taut band area containing myofascial trigger points was marked on the skin with an indelible marker and designated for electrophysiological recordings of endplate noise (EPN). All procedures of the following assessments were performed individually by two investigators who were blinded to the group assignment. 

### 4.4. Ultra-Low Frequency Transcutaneous Electrical Nerve Stimulation 

ULF-TENS was produced using a commercially therapeutic electrical stimulator of physical therapy device (ES-160, ITO Co, Tokyo, Japan). The needle electrode applied at the myofascial trigger points of the masseter muscle allows the excitation of motor nerve fibers of the trigeminal nerve, resulting in contraction of the masticatory musculature. The current used is pulsed with a frequency of 2 Hz, and each pulse has a duration of 200 microseconds and an amplitude of 1–3 mA for 15 min per treatment session for 7 consecutive days. A similar procedure was applied to the animals with sham ULF-TENS treatment, but the current dose was adjusted to 0.

### 4.5. Ultrasound-Guided Electrophysiological Recording of Endplate Noise

A two-channel digital EMG machine (Neuro-EMG-Micro; Neurosoft, 5, Voronin Str, Ivanovo, Russia) and monopolar needle electrodes (37 mm disposable Teflon-coated model) were used. The low-cut frequency filter was set at 100 Hz; and the high-cut one, at 1000 Hz. Under ultrasonic guidance (16HL7, Terason t3000™ Ultrasound System, Ormond Beach, FL, USA), the search needle for EPN recording was inserted in the direction of the taut band region with a focal hypoechoic (darker) area at an angle approximately 60 degrees to the surface of the muscle. A common reference needle electrode for each channel was placed on the incised skin. 

The procedure for measuring EPN prevalence was conducted as described previously [45]. After initial insertion, the needle was advanced very slowly under gentle rotation. Each advance was about 1 mm. When the search needle approached an active locus, continuous electrical activities of EPN could be heard. The search needle was then pressed laterally in three directions, one of which often resulted in the appearance of EPN. If not, the search needle was further advanced for a minimum distance, which might then result in appearance of EPN. A site was designated an “EPN locus” when spontaneous continuous low-voltage potentials of at least 10 μV were maintained for at least 30 s. After five advances in one direction (one track), the search needle was withdrawn to its starting point, and then redirected to penetrate unexplored muscle tissue on a second track. In total, 15 different loci along three tracks were explored in one myofascial trigger point region. 

### 4.6. Maximum Jaw-Opening Distance

The distances between upper and lower incisors of midline at the maximum mouth opening was measured using a Vernier caliper with the rat anesthetized in the neutral head and neck positions.

### 4.7. Quantitative Analysis for SP, MOR, and c-Fos Immunohistochemistry

Animals were euthanized by anesthetic overdose after treatments and their brainstems were then collected for immunohistochemical analyses. The brainstem was fixed in 4% paraformaldehyde for 2 weeks at 4 °C and then embedded in paraffin. Specimens were serially cut at 5 μm thickness in the sagittal plane with a microtome at locations from Bregma −11.60 to −9.16 mm (containing parabrachial nucleus and rostral ventromedial medulla regions). Immunohistochemical staining assay was examined in five alternate sections per brain location per rat and each specimen produced approximately 20 sections, which were selected by a systematic-random series with a random start for analysis. The sections were incubated overnight at 4 °C with antibody against SP (#20064, ImmunoStar, Hudson, WI, USA), MOR (#24216, ImmunoStar, Hudson, WI, USA), and c-Fos (#2250, Cell Signaling Technology, Danvers, MA, USA) followed by incubation with biotinylated goat anti-rabbit IgG secondary antibody (Jackson ImmunoResearch Laboratories, Inc., West Grove, PA, USA) and a streptavidin-horseradish peroxidase conjugate (Jackson ImmunoResearch Laboratories, Inc., West Grove, PA, USA). Finally, the sections were visualized as brown precipitates by adding 3,3′-diaminobenzidine (DAB, Pierce, Rockford, IL, USA) as a substrate. Sections were examined under a light microscope (BX43, Olympus America Inc., New York, NY, USA) and photographed in five randomly selected fields using a digital color camera (DP70, Olympus America Inc.). Digital images were analyzed by computer-based morphometry using the ImageScope software package with the Color Deconvolution v9 tool (v9.1.19.1571, Aperio, Vista, CA, USA). The percentages of strong positive pixels for SP-LI, MOR-LI, and cFos-LI to total area pixels of parabrachial nucleus and rostral ventromedial medulla were quantified. 

### 4.8. Statistical Analysis

All data are expressed as mean ± standard deviation (SD). The sample size of 40 animals provided a power of 0.80 with an alpha of 0.05 to detect differences among 4 groups in assessment value. The Kolmogorov–Smirnoff test indicated a non-normal distribution of all data in all measures. The effects of ULF-TENS treatment on EPN prevalence in masseter muscle, maximum interincisal distance, and the percentages of strong positive pixels in contents of SP and MOR in parabrachial nucleus as well as c-Fos in rostral ventromedial medulla were each examined with a Kruskal–Wallis test to determine the significant differences among four groups (MU, MsU, sMU, and sMsU). Post hoc comparisons between two groups were analyzed with the Mann–Whitney test. A Bonferroni adjustment was done and results were considered significant for a *p* value of 0.0083 (=0.05/6). A Friedman test was performed to determine the difference among three time points (pre-induction, pre-treatment, and post-treatment) in each group and a post-hoc analysis was conducted using Wilcoxon signed-rank test. A *p* value of <0.017 (=0.05/3, Bonferroni adjustment) was considered statistically significant. All data were analyzed using SPSS version 22.0 for Windows (SPSS Inc., Chicago, IL, USA).

## 5. Conclusions

MMP is a multifactorial disorder, mostly involving occlusal, skeletal, and psychological disturbances manifested in muscular structures. This research found increased MOR-LI and reduced SP-LI in the parabrachial nucleus and increased c-Fos-LI in the rostral ventromedial medulla, as well as improvements of jaw-opening distance and EPN prevalence after ULF-TENS treatment in the MMP animal model, indicating that MMP can be modulated by ULF-TENS. It is the first study demonstrating the underlying mechanism of ULF-TENS, which is probably beneficial to management of MMP. More biochemical studies may further reveal a central nociceptive transmission mechanism that can provide more information of such neuroplasticity alterations arising from effects of ULF-TENS on myofascial trigger points in the orofacial region. Moreover, it may be possible that ULF-TENS may provide an innovative measure against post-operative complications in various scenarios, such as orofacial pain induced by molar extraction in clinical non-invasive interventions.

## Figures and Tables

**Table 1:** The prevalence of endplate noise and maximum jaw-opening distances at each evaluation time in four groups.

		Pre-Induction	Pre-Treatment	Post-Treatment	^2^ Differences among Timepoints, *p* Value
EPN prevalence	MU	27.30 ± 5.68	52.60 ± 4.77 *^†§^	28.10 ± 6.03 ^‡^	0.00037
(%)	MsU	29.40 ± 7.07	51.20 ± 8.94 *^†§^	54.50 ± 6.17 *^†§||^	0.00038
	sMU	22.70 ± 4.64	25.30 ± 6.00	24.80 ± 4.52	0.04214 (NS)
	sMsU	28.10 ± 4.01	27.4 ± 4.76	28.20 ± 2.53	0.86687 (NS)
^1^ Differences among groups, *p* value		0.0623 (NS)	1.87 × 10^−6^	1.02 × 10^−5^	
Jaw-opening distance	MU	2.23 ± 0.16	1.92 ± 0.16 *^†§^	2.15 ± 0.08 ^‡^	0.00183
(cm)	MsU	2.21 ± 0.15	1.95 ± 0.12 *^†§^	1.93 ± 0.09 *^†§||^	0.00037
	sMU	2.16 ± 0.19	2.20 ± 0.19	2.24 ± 0.18	0.13904 (NS)
	sMsU	2.22 ± 0.12	2.20 ± 0.11	2.26 ± 0.15	0.04436 (NS)
^1^ Differences among groups, *p* value		0.88 (NS)	3.03 × 10^−5^	1.61 × 10^−4^	

Data are expressed as means ± SD. ^1^: Kruskal–Wallis test and Mann–Whitney post hoc test (*p* < 0.0083, Bonferroni adjustment). ^2^: Friedman test and Wilcoxon’s sign-rank post hoc test (*p* < 0.017, Bonferroni adjustment). *^, †, ‡^: *p* < 0.0083, indicate values compared to sMU, sMsU, and MsU groups, respectively. ^§, ||^: *p* < 0.017, indicate values compared with those of pre-induction and pre-treatment time points, respectively. NS, no significant difference.

**Table 2:** The substance P (SP), μ-opiate receptors (MOR), and c-Fos immunoreactivity in the parabrachial nucleus and rostral ventromedial medulla in four groups.

	MU	MsU	sMU	sMsU	^1^ Differences among Groups, *p* Value
Parabrachial nucleus (%)					
SP	21.18 ± 2.19 *^†‡^	49.33 ± 11.42 ^†‡^	9.89 ± 0.35	8.49 ± 2.63	*p* < 0.0001
MOR	18.63 ± 5.15 *^†‡^	2.10 ± 0.11 ^†‡^	9.43 ± 2.85	6.09 ± 3.18	*p* < 0.0001
Rostral ventromedial medulla (%)					
c-Fos	43.39 ± 10.73 *^‡^	13.19 ± 2.04 ^†‡^	26.33 ± 5.08 ^‡^	10.97 ± 1.15	*p* < 0.0001

Data are expressed as means ± SD. ^1^ Comparisons among four groups were analyzed with the Kruskal–Wallis test and a series of Mann–Whitney tests were be performed for post hoc analyses (*p* < 0.0083, Bonferroni adjustment). *^, †, ‡^: *p* < 0.0083, indicate values compared to MsU, sMU, and sMsU groups, respectively.

## Data Availability

The data that support the findings of this study are available from the corresponding author upon reasonable request.
